# Serum 25 Hydroxyvitamin D Levels During Pregnancy in Women with Asthma: Associations with Maternal Characteristics and Adverse Maternal and Neonatal Outcomes

**DOI:** 10.3390/nu12102978

**Published:** 2020-09-29

**Authors:** Megan E. Jensen, Carlos A. Camargo, Soriah M. Harvey, Peter G. Gibson, Vanessa E. Murphy

**Affiliations:** 1 Priority Research Centre Grow Up Well, Hunter Medical Research Institute and University of Newcastle, New Lambton Heights, NSW 2305, Australia; soriah.harvey@uon.edu.au (S.M.H.); vanessa.murphy@newcastle.edu.au (V.E.M.); 2 Department of Emergency Medicine, Massachusetts General Hospital, Boston, MA 02114, USA; CCAMARGO@PARTNERS.ORG; 3 Priority Research Centre for Healthy Lungs, Hunter Medical Research Institute and University of Newcastle, New Lambton Heights, NSW 2305, Australia; peter.gibson@newcastle.edu.au; 4 Department of Respiratory and Sleep Medicine, John Hunter Hospital, New Lambton Heights, NSW 2305, Australia

**Keywords:** maternal nutrition physiology, vitamin D, pregnancy, asthma, maternal obesity, gestational weight gain, infant, newborn

## Abstract

Low 25-hydroxyvitamin D (25(OH)D) levels are common in pregnancy and associated with adverse maternal/neonatal outcomes. In pregnant women with asthma, this study examined the association of lifestyle- and asthma-related factors on 25(OH)D levels and maternal/neonatal outcomes by vitamin D status. Serum 25(OH)D was measured at 16 and 35 weeks gestation in women with asthma (*n* = 103). Body mass index (BMI), gestational weight gain (GWG), smoking status, inhaled corticosteroid (ICS) use, asthma control, airway inflammation, and exacerbations, and maternal/neonatal outcomes were collected. Baseline and change (Δ) in 25(OH)D were modelled separately using backward stepwise regression, adjusted for season and ethnicity. Maternal/neonatal outcomes were compared between low (25(OH)D < 75 nmol/L at both time points) and high (≥75 nmol/L at one or both time points) vitamin D status. Fifty-six percent of women had low vitamin D status. Obesity was significantly associated with lower baseline 25(OH)D (Adj-R^2^ = 0.126, *p* = 0.008); ICS and airway inflammation were not. Excess GWG and season of baseline sample collection were significantly associated with Δ25(OH)D (Adj-R^2^ = 0.405, *p* < 0.0001); asthma-related variables were excluded (*p* > 0.2). Preeclampsia was more common in the low (8.6%) vs. high (0%) vitamin D group (*p* < 0.05). Obesity and excess GWG may be associated with gestational 25(OH)D levels, highlighting the importance of antenatal weight management.

## 1. Introduction

Maternal nutritional status is a major modifiable determinant of neonatal nutritional status and both maternal and offspring health outcomes. A low circulating level of 25 hydroxyvitamin D (25(OH)D) is a preventable, but common problem during pregnancy [1,2]. The role of vitamin D in health and disease prevention involves effects on hormonal pathways, immune system development and infection, as well as cell proliferation and differentiation [3,4]. Moreover, vitamin D is important for the growth and development of the skeletal system via calcium metabolism [3,5]. Low maternal 25(OH)D levels have also been associated with an increased risk of poor maternal and neonatal outcomes, including gestational diabetes, preeclampsia, preterm birth, and low-birth-weight infants [6,7,8], with vitamin D implicated in placental and immune function, neurodevelopment and lung development [9]. Adequate 25(OH)D levels during pregnancy are therefore necessary for optimal maternal, fetal and infant health. Neonatal vitamin D levels are largely determined by maternal vitamin D levels during pregnancy [10]. Therefore, low 25(OH)D levels during pregnancy directly affect neonatal 25(OH)D levels [11,12] and the associated health consequences.

Blood 25(OH)D levels have been shown to be low in women with asthma during pregnancy [13]. Asthma affects approximately 12% of women during pregnancy [14] and has been associated with an increased risk of adverse maternal and neonatal outcomes including preeclampsia [15], preterm birth, small-for-gestational age (SGA) infants, and neonatal hospitalization and mortality, highlighting this as a high risk group [16]. In a cohort of pregnant women with and without asthma, higher 25(OH)D levels (≥75 nmol/L) have been associated with a lower risk of preeclampsia, as well as better asthma control during pregnancy [15,17]. Furthermore, in women with asthma, low 25(OH)D levels during pregnancy have been associated with a higher prevalence of infant wheeze, acute-care presentations and oral corticosteroid (OCS) use [13], suggesting that maternal 25(OH)D levels may affect both maternal and infant health outcomes. A recent meta-analysis has also linked maternal vitamin D sufficiency during pregnancy to a decreased risk of asthma or recurrent wheeze in children whose mothers have asthma [18]. Factors influencing 25(OH)D levels in women with asthma during pregnancy require further investigation.

Several studies have examined factors associated with 25(OH)D levels in pregnancy in the general population [19,20,21,22,23,24,25,26,27,28,29,30,31,32]. Certain environmental factors have been associated with 25(OH)D levels during pregnancy including sun exposure [19], with dermal synthesis of cholecalciferol due to ultraviolet (UV) B radiation one of the main contributors to circulating 25(OH)D levels, and season of blood draw, with higher serum 25(OH)D concentrations expected in summer months [20,21,23,24,26,33]. In addition, low 25(OH)D levels are more common in non-white populations and those with higher melanin pigmentation [22,27,28,29,32], with race demonstrated to be the most important risk factor for vitamin D deficiency or insufficiency in a previous study [2]. Other factors that have been linked to low 25(OH)D levels in pregnancy include maternal smoking and alcohol use [20,23,30], lower education level [28], and low total dietary vitamin D intake and supplement use [22,27,28,31].

Body mass index (BMI) has previously been linked to lower 25(OH)D levels in the non-pregnant population [34,35], with percentage fat mass inversely related to 25(OH)D levels [36]; however, results in pregnant cohorts are less clear, with three studies showing a negative association between BMI and pregnancy 25(OH)D levels [20,37,38], one study showing a positive association [19], and four studies reporting no association [21,23,24,33]. The impact of gestational weight gain (GWG) on 25(OH)D levels is also unclear, with one study finding no association between GWG and 25(OH)D levels during pregnancy [39], a second reporting a negative association [37], and a third reporting a negative association, but only among women with pregestational overweight [25]. It is also not clear how weight status or weight gain interacts with 25(OH)D levels during pregnancy in women with asthma. We have previously demonstrated a high prevalence of overweight and obesity, and excessive GWG, in pregnant women with asthma [13,14]; therefore, examining whether weight status affects maternal 25(OH)D levels during pregnancy is particularly relevant to this group. In addition, whether asthma-related factors, namely airway inflammation, asthma control, inhaled corticosteroid (ICS) use, and exacerbations of asthma, are associated with 25(OH)D levels during pregnancy has not been examined.

Therefore, in pregnant women with asthma, the aims of this study were to: (1) explore the association between lifestyle (weight status, GWG, smoking) and asthma-related factors (airway inflammation, asthma control, ICS use, exacerbations) and 25(OH)D levels during pregnancy; and (2) compare the incidence of adverse maternal and neonatal outcomes by low vs. high vitamin D status during pregnancy.

## 2. Materials and Methods

This is a secondary analysis of data collected during the period 2007–2009 from a cohort of 168 pregnant women with asthma, aged ≥18 years, recruited between 12 and 20 weeks gestation via the John Hunter Hospital Antenatal Clinic (Newcastle, Australia [latitude 32.93 °S]) into a study of respiratory viral infection in pregnancy [40]. Concurrently, the majority (*n* = 157, 93%) of women also participated in the Managing Asthma in Pregnancy (MAP, 2007–2010) study, a RCT of monthly fractional exhaled nitric oxide (FENO)-guided asthma management versus symptoms-guided management during pregnancy (Hunter New England Human Research Ethics Committee approval # 07/02/21/3.06, Australian and New Zealand Clinical Trials Registry # 12607000561482) [41]. Written informed consent was obtained prior to enrolment in this study. Asthma was determined by self-reported physician diagnosis and recent asthma symptoms or medication use, with confirmation by a respiratory physician at enrolment. Women were excluded if they had used more than three OCS courses in the past 12 months or had another chronic lung disease. All women were followed monthly until birth.

### 2.1. Measurements

Ethnicity was self-reported at baseline. Tobacco smoke exposure was self-reported and determined objectively by measurement of exhaled carbon monoxide (≥10 ppm, piCO Smokerlyzer Breath CO Monitor, Bedfont, UK) and urinary cotinine (≥level 5 or 2840 nmol/L, Nicalert, NYMOX, Saint-Laurent, Quebec, Canada). Maternal height and weight were measured at each study visit and baseline BMI (kg/m^2^) and GWG calculated. Baseline BMI was categorized as non-overweight (<25 kg/m^2^), overweight (≥25–<30 kg/m^2^) or obese (≥30 kg/m^2^). GWG was classified as within or exceeding recommended guidelines [42]. Airway inflammation was measured via FENO (ECOMEDICS online chemiluminescence analyzer, Duernten Switzerland; 50 mL/s flow rate). Lung function was assessed by spirometry (EasyOne Spirometer, Niche Medical, North Sydney Australia), with forced expiratory volume in 1 s (FEV_1_) and forced vital capacity (FVC) reported as a percentage of their predicted values (NHANES III) [43], and the ratio documented (FEV_1_/FVC). Asthma control was assessed using the validated Asthma Control Questionnaire (ACQ) [44]. Asthma medications and exacerbations requiring medical intervention (unscheduled physician appointment, emergency department presentation, hospitalization, or OCS) were recorded prospectively by participant report. Maternal and neonatal outcomes were documented from the medical records including gestational hypertension (GH, defined as systolic blood pressure ≥140 mmHg and/or diastolic blood pressure ≥90 mmHg, which was not preexisting and developed de novo >20 weeks gestation, without organ disorders), preeclampsia (PE, defined as systolic blood pressure ≥140 mmHg and/or diastolic blood pressure ≥90 mmHg, which developed de novo >20 weeks gestation and the presence of proteinuria >20 weeks gestation), gestational diabetes mellitus (defined as a fasting blood glucose level ≥5.5 mmol/L or a 2-h blood glucose level ≥8.0 mmol/L following a 75 g glucose load, according to guidelines at the time of this study) [45], labor type, mode of birth, infant anthropometry, infant Apgar score at one and five minutes, neonatal respiratory distress and neonatal intensive care unit (NICU) admission. Gestational age was calculated from the estimated date of confinement (based on either the last menstrual period or early ultrasound) and the date of birth, with preterm birth defined as <37 weeks gestation.

A non-fasting blood sample was collected via venepuncture from a subset of participants at baseline and late pregnancy (approximately 16 and 35 weeks gestation, respectively) and stored in serum aliquots at −80 °C. Batch analysis of total 25(OH)D (comprised of 25(OH)D_2_ and 25(OH)D_3_) using enzyme-linked immunosorbent assay (Abbott Architect assay, Abbott Park, IL; intra- and interassay coefficients of variation <10%) took place at Massachusetts General Hospital (Boston, MA, USA). Season was documented by date of collection (Summer, Winter, Spring, and Autumn). The Massachusetts General Hospital core laboratory is a clinical laboratory improvement amendments-certified facility, which uses rigorous methods and continuously updated reference standards for biomarker assessment. Intra- and interassay coefficients of variation (CV) were both less than 8% for 25(OH)D. Although other metabolites may be included in the future, it is currently accepted that singular measurement of serum 25(OH)D is the biologic marker of vitamin D status clinically [34]. 25(OH)D level was dichotomized at 75 nmol/L, according to Endocrine Society guidelines for vitamin D status [34] and as used in previous studies [13]. Women were grouped by 25(OH)D level during pregnancy: (i) 25(OH)D <75 nmol/L at both time points (low) versus (ii) 25(OH)D ≥75 nmol/L at one or both pregnancy time points (high).

### 2.2. Analysis

Statistical analyses were conducted using Stata Version 11.1 (StataCorp LP, College Station, TX, USA). Continuous variables were presented as the mean ± standard deviation (SD) or [interquartile range, IQR] and analyzed using student *t* test or Wilcoxon rank-sum test, with proportions (%) analyzed using X^2^ test (demographics, maternal and neonatal outcomes). Statistical significance was set at a two-sided *p* < 0.05. Change (Δ) in 25(OH)D levels were calculated as the difference between the late and early pregnancy measure. Multiple stepwise linear regressions were performed for baseline and Δ25(OH)D as dependent variables and adjusted for ethnicity and season given the known associations between skin color and UVB exposure with 25(OH)D levels. The ∆25(OH)D was also adjusted for baseline 25(OH)D levels. Asthma-related outcomes (FENO, ACQ and ICS use at enrolment, and exacerbations during pregnancy), smoking status and BMI category at enrolment, and GWG above recommendations, were included as independent variables, with backward elimination for parameters with a *p*-value >0.2.

## 3. Results

**Figure 1:**
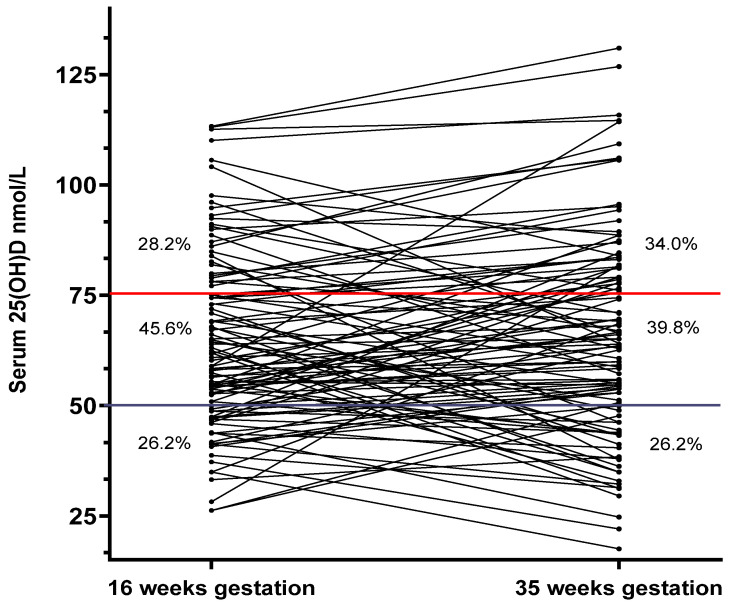
Serum 25(OH)D levels at 16 and 35 weeks gestation in pregnant women with asthma. Serum 25(OH)D at 16 and 35 weeks gestation in 103 pregnant women with asthma. Dots represent individual 25(OH)D values, with connecting lines demonstrating the within-person trajectory from 16 to 35 weeks gestation. Major horizontal lines at 75 and 50 nmol/L represent the cut-points for vitamin D ‘sufficiency’ and ‘deficiency’. The proportion of women who fell into the categories of ‘sufficient’, ‘insufficient’ and ‘deficient’ are presented on the graph at both 16 and 35 weeks gestation. 25(OH)D: 25-hydroxyvitamin D.

Baseline and late serum 25(OH)D measurements, and maternal and neonatal outcomes, were available for 103 women with asthma; all were singleton pregnancies. The majority of women were white (81.6%), with 16.5% current smokers. At enrolment, 32.0% and 40.8% of women were overweight and obese, respectively. The average absolute weight gain during pregnancy (16–35 weeks) was 7.7 ± 4.5 kg (*n* = 88), with GWG exceeding recommendations in 70.5% of women. The mean 25(OH)D level at 16 and 35 weeks was 64.77 ± 20.6 (median [IQR] 60.9 [49.2, 78.10]; range 26.20 to 113.3) nmol/L and 65.59 ± 23.46 (median [IQR] 63.90 [48.90, 81.40]; range 17.50 to 131.0) nmol/L, respectively. The change in 25(OH)D levels from 16 to 35 weeks ranged from 46.20 to 55.40 nmol/L, with an average difference of 1.33 ± 19.79 (median 5.00 [−14.20, 13.20]) nmol/L (Figure 1).

Fifty-six percent of women (*n* = 58) had 25(OH)D <75 nmol/L at both 16 and 35 weeks gestation, and 44% (*n* = 45) had 25(OH)D ≥75 nmol/L at one or both time points. Demographics are presented by vitamin D group in Table 1. There were no significant group differences by vitamin D status, with the exception of BMI, which was higher in the low (vs. high) vitamin D group. Baseline asthma control was similar between the low and high vitamin D group (ACQ: 1.2 (1.0) vs. 1.1 (0.8), *p* = 0.61), as was the proportion prescribed ICS medication (25.9% [*n* = 15] vs. 31.1% [*n* = 14], *p* = 0.56; ICS dose 800 [500, 800] vs. 800 [400, 800] mcg, *p* = 0.52). The proportion of women experiencing an asthma exacerbation during pregnancy was not significantly different between the low and high vitamin D group (41.4% [*n* = 24] vs. 35.6% [*n* = 16], *p* = 0.55).

### 3.1. Asthma- and Lifestyle-Related Variables Associated with 25(OH)D Levels in Pregnancy

Obesity was associated with a significantly lower 25(OH)D level at 16 weeks gestation, compared to a BMI <25 kg/m^2^, after controlling for season of blood collection and ethnicity (Table 2); however, airway inflammation (measured by FENO) and ICS use were not associated with baseline 25(OH)D levels. Smoking and ACQ score were not retained in the model of baseline 25(OH)D (*p* > 0.2).

Only baseline weight status and GWG were retained in the model of ∆25(OH)D, controlling for ethnicity and season; asthma-related variables (FENO, ACQ, ICS use, exacerbations during pregnancy), smoking status and baseline 25(OH)D ≥75 nmol/L were not retained in the model due to a *p*-value >0.2. Baseline sample collection in Autumn was associated with a decrease, while sample collection in Winter or Spring were associated with an increase, in 25(OH)D levels from 16 weeks to 35 weeks gestation (Table 2). Excessive GWG was associated with a statistically significant decline in 25(OH)D levels from 16 to 35 weeks gestation.

### 3.2. Maternal Vitamin D Status and Maternal and Neonatal Outcomes

The incidence of preeclampsia was significantly higher in those with 25(OH)D levels <75 nmol/L during pregnancy, compared with 25(OH)D levels ≥75 nmol/L (Table 3). There were no eclamptic cases in either group. There were no miscarriages; however, one woman in the low vitamin D group, delivered a stillborn infant. There was a clinically important difference in NICU admissions (*p* = 0.26) and neonate respiratory distress (*p* = 0.10) between the low and high vitamin D groups, but this was not statistically significant. There were no statistically significant differences for other maternal or neonatal outcomes by vitamin D status.

## 4. Discussion

This was the first study to report on factors associated with 25(OH)D levels during pregnancy in women with asthma in the Australian context. In predominantly white women of European descent with mild asthma, low vitamin D status was common during pregnancy. Both maternal obesity and GWG above guideline recommendations, regardless of BMI category at enrolment, were significant modifiable factors associated with 25(OH)D levels during pregnancy; however, asthma-related variables were not associated with 25(OH)D levels in this group of women. These results provide evidence to support the importance of early nutrition intervention in pregnant women with asthma.

Our results highlight the importance of achieving a healthy BMI prior to pregnancy and maintaining GWG within recommendations, regardless of BMI category. Obesity, but not overweight, was significantly associated with lower early–mid pregnancy 25(OH)D levels in this group of women with asthma. This is in agreement with a 2011 study conducted in Australia and New Zealand that found BMI had a significant negative association with serum 25(OH)D levels in two general pregnancy cohorts; those with a BMI ≥30 kg/m^2^ were three times more likely to have suboptimal 25(OH)D levels, compared to those who had a BMI <30 kg/m^2^ (adjusted odds ratio [aOR] 3.0, 95%CI 1.4, 4.3) [27]. BMI was also found to be negatively associated with 25(OH)D levels at 15 weeks gestation in an Irish population (adjusted mean difference −0.3, 95%CI −0.5, −0.04) [38]. This is supported by a Swedish study reporting a weak, but significant, association between both higher pregestational BMI (OR 1.10, 95%CI 1.01, 1.20) and BMI during pregnancy (OR 1.10, 95%CI 1.02, 1.21) and vitamin D deficiency in the first trimester (<50 nmol/l) [20].

In our group of pregnant women with asthma, GWG above recommendations was also negatively associated with the change in 25(OH)D level from 16 to 35 weeks gestation, which has not been previously examined in women with asthma. This is in agreement with a previous study by Moon et al. in 1753 women demonstrating a significant decrease in 25(OH)D levels over pregnancy with greater weight gain [37]. However, these results contrast a second study which found no association, albeit in a much smaller sample size (*n* = 237) [39]. A third study from Figueiredo et al. found an association between maternal weight gain and 25(OH)D levels but this was limited to women with pregestational overweight (*n* = 163) [25]. The association between excess weight and lower 25(OH)D levels may be attributable to adipose tissue sequestration, or volumetric dilution, of endogenous and exogenous vitamin D [46]. Low vitamin D intake from poor diet and inadequate sun exposure associated with a sedentary lifestyle, notably minimal outdoor activity, is another possible explanation [46]. Our results and the previous literature highlight the role of both prenatal weight status and GWG in influencing 25(OH)D levels during pregnancy, an important factor in both maternal and neonatal health outcomes. Given the high prevalence of obesity and excessive GWG in women, including women with asthma, this area warrants further research into the impacts on maternal and neonatal nutritional health.

Smoking status was also examined in our cohort, with current smoking status not associated with either baseline, or change in, 25(OH)D levels in our group of women with asthma. With the exception of one study in a pregnant Spanish cohort [47], our results contrast the majority of previous studies demonstrating that maternal smoking is associated with low 25(OH)D levels during pregnancy [28,29,38,48]. The prevalence of smoking was similar across studies, so this may be due to our smaller sample size.

There are several other potentially important lifestyle variables that have been associated with pregnancy 25(OH)D levels, which were not available in the present study, nor did we have data on preexisting hypertension in this cohort; these are limitations of this study. Most notably, dietary and supplemental intake were not collected in our cohort, nor was a measure of sun exposure or socio-economic status, and therefore we were unable to account for these variables in our analysis. High vitamin D dietary intake [22,31] and supplementation during pregnancy [22,27], as well as outdoor recreational walking ≥4 times per week (proxy for sun exposure) [38], have been associated with higher 25(OH)D levels during pregnancy. Similarly, low sun exposure time is also a documented risk factor for vitamin D deficiency during pregnancy in a predominantly veiled and dark skinned population in Australia [49]. Therefore, future research in this area would benefit from comprehensively documenting dietary, supplemental and environmental sources of vitamin D.

Previous studies have looked at the association of various factors on 25(OH)D levels in the general population, but not specifically the association of asthma-related variables. Airway inflammation, asthma control, ICS use, and exacerbations were not associated with 25(OH)D levels at 16 weeks gestation, or the change in 25(OH)D levels from 16 to 35 weeks gestation, in this group of women. Indeed, we did not detect a difference in airway inflammation, asthma control or ICS use at enrolment (approximately 16 weeks gestation), or exacerbations requiring medical intervention during pregnancy, by vitamin D status. However, we may have lacked the power to detect a statistically significant difference in such outcomes. Furthermore, the fact that our population had relatively mild asthma may explain why there was no association between asthma-related variables and 25(OH)D levels in this group of pregnant women. Therefore, future work exploring the relationship between asthma and 25(OH)D levels in a larger sample of pregnant women with varying degrees of asthma severity is warranted.

A statistically significant higher incidence of preeclampsia was detected in women who had low (vs. high) 25(OH)D levels during pregnancy; in fact, no women in the high vitamin D group developed preeclampsia. This is in agreement with a secondary analysis of a RCT of vitamin D supplementation during pregnancy, which reported lower preeclampsia rates in women (40% with a self-reported history of physician-diagnosed asthma) with 25(OH)D levels ≥75 nmol/L in early and late pregnancy (10–18 and 32–38 weeks), compared with those who were insufficient at both time points (2.3 vs. 11.9%) [17]. Although previous meta-analyses have found an association between preterm birth and low birth weight in women with vitamin D deficiency (<50 nmol/L) during pregnancy [50,51], we did not detect a difference in these outcomes in our study. Of interest, we did observe a clinically important difference in NICU admissions and neonatal respiratory distress between the low and high vitamin D groups; however, this was not of statistical significance. This has not been examined in previous studies specifically including pregnant women with asthma, and warrants further investigation.

This study was a secondary analysis of a prospective trial, and thus not primarily designed for the outcomes of interest (i.e., factors associated with gestational 25(OH)D levels and the incidence of adverse maternal and neonatal outcomes by vitamin D status). Nevertheless, this is the first study to examine the effect of lifestyle- and asthma-related factors on 25(OH)D levels during pregnancy, in an Australian context, in a well-defined sample of women with mild asthma. Both obesity and maternal weight gain are modifiable factors that may be associated with 25(OH)D levels in pregnancy. Considering the high prevalence of obesity and GWG in women with asthma [14], this further highlights the importance of dietary intervention in this group of women in the antenatal period. A larger sample size would provide increased power to further examine the association of lifestyle, and disease-related, factors on maternal 25(OH)D levels. Moreover, given that women with indications of more severe asthma, e.g., recent OCS use, were excluded from the trial, it is unclear whether the associations observed would be different if studied in women with more moderate to severe disease. Furthermore, our results suggest that further investigation of the effect of maternal vitamin D status on maternal and neonatal outcomes in women with asthma is warranted. Nutritional therapy to improve vitamin D status may be an acceptable adjunct therapy to the clinical management of pregnant women with asthma and requires further investigation.

## Figures and Tables

**Table 1:** Demographics by maternal vitamin D status during pregnancy for women with asthma.

Variable	25(OH)D <75 nmol/L (*n* = 58)	25(OH)D ≥75 nmol/L (*n* = 45)	*p*-Value
Age, years	28.4 (5.5)	28.7 (5.9)	0.77
Parity, *n*	1 [0, 1]	1 [0, 1]	0.83
Ethnicity: European, *n* (%)	48 (82.8%)	36 (80.0%)	0.72
Smoking during pregnancy, *n* (%)	9 (15.5%)	8 (17.8%)	0.76
Preexisting diabetes, *n* (%)	3 (5.3%)	1 (2.2%)	0.28
Body mass index, kg/m^2^	30.6 (7.8)	27.2 (5.3)	**0.01**
Overweight and obese, %	46 (79.3%)	29 (65.9%)	0.13
Gestational weight gain (16–35 weeks gestation), kg	8.0 (4.6)	7.3 (4.2)	0.45
Weight gain per week above recommendations, *n* (%)	36 (72%)	26 (68.4%)	0.72
FENO, ppb	14.9 [6.7, 29.8]	14.4 [5.9, 31.5]	0.99
FEV_1_, %predicted	93.7 (15.6)	93.1 (15.9)	0.86
FVC, %predicted	105.0 (2.3)	101.3 (17.7)	0.30
FEV_1_/FVC, %	77.8 (7.8)	80.3 (7.2)	0.14

FENO, fractional exhaled nitric oxide. FEV_1_, forced expiratory volume in 1 s. FVC, forced vital capacity. Bolded *p*-values are <0.05.

**Table 2:** Asthma- and lifestyle-related variables associated with 25(OH)D levels in pregnant women with asthma.

	Baseline 25(OH)D		Δ 25(OH)D	
Final Model	*n* = 100, Adj-R^2^ = 0.126, *p*-Value = 0.008		*n* = 86, Adj-R^2^ = 0.405 *p*-Value <0.0001	
Variable	Coefficient (95% CI)	*p*-Value	Coefficient (95% CI)	*p*-Value
Ethnicity: European *	1.18 (−9.42, 11.77)	0.83	2.15 (−7.28, 11.58)	0.65
Season: baseline sample collection *				
Autumn	−2.77 (−15.14, 9.59)	0.66	−11.89 (−22.62, −1.16)	**0.03**
Winter	−9.74 (−21.24, 1.75)	0.10	14.28 (4.64, 23.92)	**0.004**
Spring	−5.56 (−17.88, 6.75)	0.37	16.79 (5.97, 27.60)	**0.003**
BMI category: baseline				
Overweight	−6.81 (−16.91, 3.29)	0.18	5.17 (−3.83, 14.16)	0.26
Obese	−13.70 (−23.48, −3.91)	**0.007**	8.64 (−0.06, 17.35)	0.051
Baseline FENO, ppb	0.10 (−0.03, 0.23)	0.13	-	
ICS use	7.42 (−1.56, 16.41)	0.1	-	
Excessive gestational weight gain	NA		−7.77 (−15.48, −0.05)	**0.048**

25(OH)D, 25-hydroxy vitamin D; Δ25(OH)D, change in 25(OH)D from 16 to 35 weeks gestation; BMI, body mass index; FENO, fractional exhaled nitric oxide; ICS, inhaled corticosteroids; NA, variable non-applicable to model therefore not included. ‘-’ variable excluded from regression model in backward elimination (*p* > 0.2). * variables ethnicity and season forced into both models. Bolded *p*-values are <0.05.

**Table 3:** Maternal and neonatal outcomes by maternal vitamin D status during pregnancy for women with asthma.

Variable	25(OH)D < 75 nmol/L (*n* = 58)	25(OH)D ≥ 75 nmol/L (*n* = 45)	*p*-Value
Gestational hypertension	4 (6.9%)	4 (8.9%)	0.71
Preeclampsia, *n* (%)	5 (8.6%)	0 (0%)	**0.04**
Gestational diabetes, *n* (%)	1 (1.8%)	1 (2.2%)	0.88
*Labor type*			
Spontaneous	34 (58.6%)	27 (60.0%)	0.89
Induced	16 (27.6%)	11 (24.4%)	0.72
Spontaneous and augmented	1 (1.7%)	0 (0)	0.38
Vaginal birth, *n* (%)	44 (75.9%)	35 (77.8%)	0.82
Gender: male, *n* (%)	28 (48.3%)	21 (46.7%)	0.87
Gestational age at birth, weeks	39.7 (1.3)	39.5 (1.6)	0.27
Preterm birth, *n* (%)	3 (5.2%)	3 (6.7%)	0.75
Birth weight, grams	3498 (591)	3370 (578)	0.14
Birth length, cm	51.3 (2.9)	51.2 (2.7)	0.43
Birth head circumference, cm	34.3 (1.9)	34.0 (1.8)	0.23
Apgar 1, score	9 [7, 9]	9 [7.5, 9]	0.84
Apgar 5, score	9 [9, 9]	9 [9, 9]	0.87
NICU admission, *n* (%)	6 (10.5%)	2 (4.4%)	0.26
Respiratory distress, *n* (%)	6 (10.5%)	1 (2.2%)	0.10

NICU, neonatal intensive care unit. Bolded *p*-values are <0.05.
